# The FunAqua dataset of global fungal biodiversity in aquatic ecosystems

**DOI:** 10.1038/s41597-026-07729-8

**Published:** 2026-09-24

**Authors:** V. Prins, L. Tedersoo, V. Mikryukov, P. Paiste, M. Sepp, H. Grossart, V. Kisand, A. Laas, H. Tammert, Kessy Abarenkov, A. Agan, H. Agasild, R. Agha, J. Alatalo, J. Alvarez-Manjarrez, A. Ameryk, J. Anderson, S. Anslan, A. Antão-Geraldes, A. Antosiak, J. Arce Funck, R. Arias-Real, M. Ariyan, M. Bahram, S. Bansal, R. Bao, S. Beck, P. Bernotas, N. Berry, S. Bertilsson, C. Birnbaum, M. Bonk, A. V. Borges, F. Botez, F. Q. Brearley, J. Brookes, D. Bruno, A. Budzyńska, G. Bullerjahn, M. Bundschuh, B. Calheiros-Nogueira, R. Calore, C. Capelli, L. Caputo, R. Carballeira, I. Chronis, F. Čiampor, Z. Čiamporová-Zaťovičová, D. Craig, Z. Csabai, N. Cukrov, L. da Silva, E. de Eyto, J. Delgado, I. Dimante-Deimantovica, I. Domaizon, R. Dondajewska-Pielka, T. Dornan, R. Drenkhan, K. Drouillard, S. Duarte, O. Dulya, A. Dzhulai, D. Dziga, B. Egeter, M. Espenberg, S. Färkkilä, S. Fazi, A. Feckler, E. Fenoy, I. Fernandes, S. Ferreira, V. Ferreira, T. Fleituch, R. Fornaroli, A. Freiwald, T. Frenken, P. Gaffney, O. García-Oliva, H. Geara, S. Gkelis, D. Gohar, R. Gołdyn, F. Grandjean, A. Gsell, C. Gutiérrez-Cánovas, P. Haase, N. Hagh-Doust, T. Harris, A. Hashem, S. Havens, B. Heidari, S. Higgins, M. Hosseyni Moghaddam, A. Ibrahim, D. Jerinkić, S. Jones, M. Kagami, S. Kahar, K. Kangro, K. Kariman, M. Kataržytė, S. Kepfer-Rojas, H. Khan, L. B. Knoll, A. Knorrn, U. Kõljalg, D. Konstantinou, J. Kotta, K. Kowalczewska-Madura, A. Kozak, B. Kulawig, J. Kupagme, I. Kušan, R. Laarmaa, P. Laas, S. Langenheder, A. Lanzén, A. Lateef, M. Ligi, T. Löffler, U. Lortou, K. Lujza, T. MacConnell, I. Maček, P. Macreadie, K. Maileht, F. Marazzi, L. Marcello, S. Markovskaja, A. Martin, S. Martínez, V. Mata, N. Matočec, R. McKay, K. McKindles, M. Mehrshad, M. Menéndez, N. Merino, A. Mešić, S. Moctar, M. Moza, P. Nõges, T. Nõges, R. O’Hanlon, B. Öğlü, J. Oja, W. Okello, H. Orav-Kotta, P. Orr, I. Osemwegie, J. Padisák, V. Pajunen, M. Panou, A. Papatheodoulou, M. Pavlovska, J. Pearman, H. Pehlak, X. Peng, A. Pereira, R. Pereira, B. Pernecker, F. Picazo, S. Pinnow, P. Pochekutova, S. Põlme, X. Pontevedra-Pombal, A. Pošta, Y. Prekrasna-Kviatkovska, J. Pruuli, M. Pruuli, T. Pruuli, N. Radoja, S. Rahimlou, R. Rannap, S. Rasconi, A. Rasmussen, W. Remmers, L. Reyes, G. Rhodes, C. Roe, O. Rojas-Castillo, T. Roslin, K. Runnel, J. Rusch, M. Rybak, P. Rychtecký, S. Sahadevan, A. Saitta, A. Salehi-Najafabadi, I. Santi, J. Sarapuu, R. B. Schäfer, A. Schneeweiss, B. Scholz, G. Selmeczy, M. Smederevac-Lalić, R. Sommaruga, J. Stetler, E. Stoica, S. Stoll, D. Strand, M. Tamm, K. Tapolczai, J. Tarand, S. Teurlincx, J. Thompson, G. Thomson-Laing, L. Tiirmann, Z. Tkalčec, I. Trbojević, S. Trevathan-Tackett, N. Tsiarta, A. Tuvikene, L. Tuvikene, M. Vacaflores-Argandoña, T. Vahter, K. Vaino, A. L. Val, M. Vandergoes, A. Vasemägi, A. Vask, M. Vasquez, J. Veríssimo, J. Vesamäki, L. Virta, P. Visser, A. Viza, T. Vrålstad, C. S. Ward, P. Waryszak, J. Wierenga, P. Wilson, S. Wood, A. Woźniczka, C. Wurzbacher, P. Zingel, P. Znachor, T. E. E. dela Cruz, K. Panksep

**Affiliations:** 1 Institute of Technology, Faculty of Science and Technology, University of Tartu, Tartu, Estonia; 2 Mycology and Microbiology Center, University of Tartu, Tartu, Estonia; 3 Department of Zoology, College of Science, King Saud University, Riyadh, Saudi Arabia; 4 Department of Botany, Institute of Ecology and Earth Sciences, University of Tartu, Tartu, Estonia; 5 Department of Geology, Institute of Ecology and Earth Sciences, University of Tartu, Tartu, Estonia; 6 Chair of Hydrobiology and Fisheries, Institute of Agricultural and Environmental Sciences, Estonian University of Life Sciences, Tartu, Estonia; 7 Plankton and Microbial Ecology, Leibniz Institute of Freshwater Ecology and Inland Fisheries (IGB), Stechlin, Germany; 8 Institute of Biochemistry and Biology, Potsdam University, Potsdam, Germany; 9 University of Tartu Natural History Museum and Botanical Garden, Tartu, Estonia; 10 Chair of Silviculture and Forest Ecology, Institute of Forestry and Engineering, Estonian University of Life Sciences, Tartu, Estonia; 11 Department of Evolutionary and Integrative Ecology, Leibniz Institute of Freshwater Ecology and Inland Fisheries (IGB), Berlin, Germany; 12 Environmental Science Center, Qatar University, Doha, Qatar; 13 Instituto de Biología, Universidad Nacional Autónoma de México. Tercer circuito interior s/n, Mexico City, Mexico; 14 Department of Fisheries Oceanography and Marine Ecology, National Marine Fisheries Research Institute, Gdynia, Poland; 15 Department of Aquatic Sciences and Assessment, Division of Microbial Ecology, Swedish University of Agricultural Sciences, Uppsala, Sweden; 16 IUCN Species Survival Commission, IUCN SSC Aquatic Fungi Specialist Group, Gland, Switzerland; 17 Department of Biological and Environmental Science, Faculty of Mathematics and Science, University of Jyväskylä, Jyväskylä, Finland; 18 Centro de Investigação de Montanha (CIMO), LA SusTEC, Instituto Politécnico de Bragança, Bragança, Portugal; 19 Laboratory of Metabolomics, Faculty of Biochemistry, Biophysics and Biotechnology, Jagiellonian University, Kraków, Poland; 20 Department of Life Sciences, MARE - Marine and Environmental Sciences Centre, ARNET – Aquatic Research Network, University of Coimbra, Coimbra, Portugal; 21 Biogeoquímica y Ecología Microbiana, Museo Nacional de Ciencias Naturales, Consejo Superior de Investigaciones Científicas (MNCN-CSIC), Madrid, Spain; 22 Department of Ecology, Swedish University of Agricultural Sciences, Uppsala, Sweden; 23 Department of Agroecology, Aarhus University, Slagelse, Denmark; 24 Northern Prairie Wildlife Research Center, U.S. Geological Survey, Jamestown, USA; 25 GRICA-BIOpast Group, Centro Interdisciplinar de Química e Bioloxía (CICA), Faculty of Sciences, Universidade da Coruña, A Coruña, Spain; 26 Institute for Biodiversity and Freshwater Conservation, University of the Highlands and Islands, Inverness, Scotland; 27 Great Lakes Science Center, Tunison Laboratory of Aquatic Science, U. S. Geological Survey, Cortland, USA; 28 Department of Biology, Miami University, Oxford, USA; 29 Department of Aquatic Sciences and Assessment, Swedish University of Agricultural Sciences, Uppsala, Sweden; 30 School of Life and Environmental Sciences, Deakin University, Burwood, Australia; 31 School of Agriculture and Environmental Science, The University of Southern Queensland, Toowoomba, Australia; 32 Institute of Nature Conservation, Polish Academy of Sciences, Kraków, Poland; 33 Freshwater and oceanic science unit of research (FOCUS), Chemical oceanography Unit, University of Liège, Liège, Belgium; 34 Department of Systems Ecology and Sustainability, University of Bucharest, Bucharest, Romania; 35 Department of Natural Sciences, Manchester Metropolitan University, Manchester, UK; 36 School of Biological Sciences, Faculty of Science, Engineering and Technology, The University of Adelaide, Adelaide, Australia; 37 Centro de Investigación e Innovación y Agroambiental (CIAGRO), Universidad Miguel Hernández de Elche, Elche, Spain; 38 Department of Water Protection, Faculty of Biology, Adam Mickiewicz University in Poznan, Poznan, Poland; 39 Center for Great Lakes and Watershed Studies, Bowling Green State University, Bowling Green, USA; 40 Natural and Environmental Sciences, Rheinland-Pfälzische Technische Universität Kaiserslautern-Landau DE, Landau, Germany; 41 Institute of Microbiology, University of Applied Sciences and Arts of Southern Switzerland (SUPSI), Mendrisio, Switzerland; 42 Department of Evolutionary Biology and Environmental Studies, University of Zurich, Zurich, Switzerland; 43 Department of Environment Constructions and Design, Institute of Earth Sciences, University of Applied Sciences and Arts of Southern Switzerland, Mendrisio, Switzerland; 44 Instituto de Ciencias Marinas y Limnológicas UACh, Universidad Austral de Chile, Valdivia, Chile; 45 Departamento de Ciencias Farmacéuticas y de la Salud, Facultad de Farmacia, Universidad San Pablo-CEU, CEU Universities, Boadilla del Monte, España; 46 School of Biology, Department of Botany, Aristotle University of Thessaloniki, Thessaloniki, Greece; 47 Department of Biodiversity and Ecology, Plant Science and Biodiversity Centre, Slovak Academy of Sciences, Bratislava, Slovakia; 48 Plant Pathology, Plant Health and Integrated Pest Management, Agri-Food and Biosciences Institute, Belfast, Northern Ireland; 49 Department of Hydrobiology, University of Pécs, Pécs, Hungary; 50 Division for Marine and Environmental Research, Ruđer Bošković Institute, Zagreb, Croatia; 51 CIBIO, Centro de Investigação em Biodiversidade e Recursos Genéticos, InBIO Laboratório Associado, Universidade do Porto, Porto, Portugal; 52 BIOPOLIS Program in Genomics, Biodiversity and Land Planning, CIBIO, Vairão, Portugal; 53 Marine Institute, Newport, Ireland; 54 Centro de Innovación Tecnolóxica en Edificación e Enxeñería Civil (CITEEC), Universidade da Coruña, Coruña, Spain; 55 Institute of Food Safety, Animal Health and Environment “BIOR”, Riga, Latvia; 56 INRAE, CARRTEL, Université Savoie Mont Blanc, Thonon les Bains, France; 57 Department of Water Protection, Faculty of Biology, Adam Mickiewicz University in Poznan, Poznań, Poland; 58 Great Lakes Institute for Environmental Research, University of Windsor, Windsor, Canada; 59 Centre of Molecular and Environmental Biology (CBMA) & ARNET - Aquatic Research Network, University of Minho, Braga, Portugal; 60 Institute of Science and Innovation for Bio-Sustainability (IB-S), University of Minho, Braga, Portugal; 61 Oceanography, Graduate School of Oceanography, University of Rhode Island, Narragansett, USA; 62 Department of Geography, Institute of Ecology and Earth Sciences, University of Tartu, Tartu, Estonia; 63 University of Tartu, Tartu, Estonia; 64 Water Research Institute (IRSA-CNR), National Research Council, Montelibretti, Roma, Italy; 65 Department of Biology and Geology, University of Almería, La Cañada de San Urbano, Spain; 66 Department of Functional and Evolutionary Ecology, Estación Experimental de Zonas Áridas (EEZA), Consejo Superior de Investigaciones Científicas (CSIC), La Cañada de San Urbano, Spain; 67 Department of Environment Constructions and Design, Institute of Microbiology, University of Applied Sciences and Arts of Southern Switzerland, Bellinzona, Switzerland; 68 IUCN SSC Aquatic Fungi Specialist Group, IUCN Species Survival Commission, IUCN, Rue Mauverney 28, Gland, Switzerland; 69 Department of Earth and Environmental Sciences – DISAT, University of Milano-Bicocca, Milan, Italy; 70 Department for Marine Research, Senckenberg am Meer, Wilhelmshaven, Germany; 71 Cluster Nature & Society, HAS green academy, s-Hertogenbosch, The Netherlands; 72 Key Laboratory of the Water Cycle and Related Land Surface Processes, Institute of Geographic Sciences and Natural Resources Research, Chinese Academy of Sciences, Beijing, China; 73 Ecosystem modelling, Institute of Coastal Systems - Analysis and Modeling, Helmholtz-Zentrum hereon, Geesthacht, Germany; 74 Centro de estudios Atitlan, Universidad del Valle de Guatemala, Guatemala city, Guatemala; 75 Institute of Ecology and Earth Sciences, University of Tartu, Tartu, Estonia; 76 Department of Botany and Plant Pathology, Oregon State University, Corvallis, USA; 77 Laboratoire Ecologie Evolution Symbiose, Université de Poitiers, Poitiers, France; 78 Department of Environmental Biology, Institute of Environmental Sciences (CML), Leiden University, Leiden, The Netherlands; 79 Instituto de Investigación en Cambio Global (IICG-URJC), Universidad Rey Juan Carlos, Móstoles, Spain; 80 Department of River Ecology and Conservation, Senckenberg Research Institute and Natural History Museum Frankfurt, Gelnhausen, Germany; 81 Department of River and Floodplain Ecology, University of Duisburg-Essen, Essen, Germany; 82 Kansas Biological Survey & Center for Ecological Research, University of Kansas, Lawrence, USA; 83 Botany and Microbiology Department, Al-Azhar University, Cairo, Egypt; 84 Experimental Lakes Area, International Institute of Sustainable Development, Winnipeg, Canada; 85 IKBM, Department of Chemistry, Bioscience and Environmental Engineering, University of Stavanger, Stavanger, Norway; 86 Fishery company, Ilorin, Nigeria; 87 Department of algology and mycology, Institute of Botany and Botanical Garden “Jevremovac”, Faculty of Biology, University of Belgrade, Belgrade, Serbia; 88 Institute for Multidisciplinary Sciences, Yokohama National University, Yokohama, Japan; 89 Chair of Aquaculture, Estonian University of Life Sciences, Tartu, Estonia; 90 Department of Remote Sensing, Tartu Observatory, University of Tartu, Tõravere, Estonia; 91 UWA School of Agriculture and Environment, The University of Western Australia, Perth, Australia; 92 Marine Research Institute, Klaipėda University, Klaipėda, Lithuania; 93 Geosciences and Natural Resource Management, University of Copenhagen, Frederiksberg, Denmark; 94 Department of Marine Research, Senckenberg am Meer, Wilhelmshaven, Germany; 95 Estonian Marine Institute, University of Tartu, Tallinn, Estonia; 96 Department of Water Protection, Adam Mickiewicz University, Poznań, Poland; 97 Senckenberg Society for Nature Research, The Rhine-Main-Observatory, Frankfurt am Main, Germany; 98 Laboratory for Biological Diversity, Ruđer Bošković Institute, Zagreb, Croatia; 99 Estonian Environmental Research Centre, Tartu, Estonia; 100 Department of Ecology and Genetics/Limnology, Uppsala University, Uppsala, Sweden; 101 Marine Research, AZTI, Pasaia, Spain; 102 IKERBASQUE - Basque Foundation for Science, Bilbao, Spain; 103 Department of Plant Biology, Faculty of Life Sciences, University of Ilorin, Ilorin, Nigeria; 104 Department of Remote Sensing, Estonian Marine Institute, University of Tartu, Tallinn, Estonia; 105 Faculty of Biology and Geology, Laboratory of Advanced Hydrobiology and Biomonitoring, Babeș-Bolyai University, Cluj-Napoca, Romania; 106 Natural Sciences, College of Arts and Sciences, Colby-Sawyer College, New London, USA; 107 Water Quality Lab, Lake Sunapee Protective Association, Sunapee, USA; 108 Department of Biology, Biotechnical Faculty, University of Ljubljana, Ljubljana, Slovenia; 109 Centre for Nature Positive Solutions, Biology Department, School of Science, RMIT University, Melbourne, Australia; 110 Dept. of Earth and Environmental Sciences -DISAT, University of Milano - Bicocca, Milan, Italy; 111 Biomathematics and Statistics Scotland (JHI), Invergowrie, Dundee, United Kingdom; 112 Institute for Biodiversity and Freshwater Conservation (IBFC), Inverness College, University of the Highlands and Islands, Inverness, United Kingdom; 113 Laboratory of Mycology, State Scientific Research Institute Nature Research Centre, Vilnius, Lithuania; 114 Laboratorio de Patología Vegetal, INIA Treinta y Tres, Treinta y Tres, Uruguay; 115 Biological Sciences, Bowling Green State University, Bowling Green, USA; 116 Biology Department, Baylor University, Waco, USA; 117 Center for Reservoir and Aquatic Systems Research, Waco, USA; 118 Department of Ecology and Genetics, Limnology and Science for Life Laboratory, Uppsala University, Uppsala, Sweden; 119 University of Barcelona, Barcelona, Spain; 120 Glenn T. Seaborg Institute, Physical and Life Sciences Directorate, Lawrence Livermore National Laboratory, Livermore, USA; 121 Department of Bioecology and Aquaculture, Laboratory of Marine Ecology, Institut Mauritanien de Recherches Océanographiques et de Pêches (IMROP), Nouadhibou, Mauritania; 122 Department of Botany and Microbiology/ICUB, Faculty of Biology, University of Bucharest, Bucharest, Romania; 123 Applied Mycology and Biology Investigations Research Center, Moza Foundation, Bucharest, Romania; 124 Plant Science, Department of Agriculture, Food and the Marine, Celbridge, Ireland; 125 Soroti University, Arapai, Uganda; 126 Australian Rivers Institute, Griffith University, Griffith, Australia; 127 Department of Ecology and Natural Resources Management, Center for Development Research (ZEF), University of Bonn, Bonn, Germany; 128 Research Group of Limnology, Centre of Natural Sciences, University of Pannonia, Veszprém, Hungary; 129 Department of Geosciences and Geography, University of Helsinki, Helsinki, Finland; 130 Nature Conservation Unit, Frederick University, Lefkosia, Cyprus; 131 Department of Biology and Ecology, National Antarctic Scientific Center, Kyiv, Ukraine; 132 Cawthron Institute, Nelson, New Zealand; 133 Chair of Biodiversity and Nature Tourism, Institute of Agricultural and Environmental Sciences, Estonian University of Life Sciences, Tartu, Estonia; 134 OÜ Xenus, Koguva, Estonia; 135 School of Earth, Ocean and Environment, University of South Carolina, Columbia, USA; 136 Marine Science Institute, University of California, Santa Barbara, USA; 137 Polytechnic Institute of Bragança, Bragança, Portugal; 138 Department of Ecology, Water Institute/Research Unit Modelin Nature, University of Granada, Granada, Spain; 139 Department of Plankton and Microbial Ecology, Leibniz Institute of Freshwater Ecology and Inland Fisheries, Stechlin, Germany; 140 Departamento de Edafoloxía e Química Agrícola, Faculty of Biology, University of Santiago de Compostela, Santiago de Compostela, Spain; 141 Aiglon College, Chesières, Switzerland; 142 Käsmu Maritime School, Käsmu, Estonia; 143 GoGroup Estonia, Tallinn, Estonia; 144 Public Health Institute of the Republic of Srpska, Banja Luka, Bosnia and Herzegovina; 145 Department of Plant Pathology and Environmental Microbiology, Pennsylvania State University, Pennsylvania State College, USA; 146 Department of Zoology, Institute of Ecology and Earth Sciences, University of Tartu, Tartu, Estonia; 147 UMR CARRTEL, INRAE, Université Savoie Mont Blanc, Thonon les Bains, France; 148 Earth System Science Department, Stanford University, Stanford, USA; 149 Department of Environmental Planning and Environmental Technology, Environmental Campus Birkenfeld, Trier University of Applied Sciences, Birkenfeld, Germany; 150 Laboratories and Workshops, Earth Sciences New Zealand, Wellington, New Zealand; 151 Lancaster Environment Centre, Centre for Ecology and Hydrology, Lancaster, UK; 152 Department of Biology, Freshwater biology section, University of Copenhagen, Copenhagen, Denmark; 153 Department of Ecology, Swedish University of Agricultural Sciences (SLU), Uppsala, Sweden; 154 Ecosystems and Environment Research Programme, Faculty of Biological and Environmental Sciences, University of Helsinki, Helsinki, Finland; 155 Norwegian Veterinary Institute, Ås, Norway; 156 Department of Aquatic Microbial Ecology, Institute of Hydrobiology, Biology Centre of the Czech Academy of Sciences, České Budějovice, Czechia; 157 Department of Agricultural, Food and Forest Sciences, University of Palermo, Palermo, Italy; 158 Department of Microbiology, School of Biology, College of Science, University of Tehran, Tehran, Iran; 159 Institute of Marine Biology, Biotechnology and Aquaculture (IMBBC), Hellenic Centre for Marine Research (HCMR), Heraklion, Greece; 160 Institute of Oceanography, Hellenic Centre for Marine Research (HCMR), Heraklion, Greece; 161 Estonian Museum of Natural History, Tallinn, Estonia; 162 Ecotoxicology, Faculty of Biology, University of Duisburg-Essen, Essen, Germany; 163 Ecotoxicology, Research Centre One Health, Research Alliance Ruhr, Essen, Germany; 164 German Environment Agency, Dessau-Roßlau, Germany; 165 Marine Biotechnology, BioPol ehf IS, Skagaströnd, Iceland; 166 Faculty of Natural Resource Sciences, School of Health, Business and Natural Sciences, University of Akureyri, Akureyri, Iceland; 167 Research Group of Limnology, Center for Natural Science, University of Pannonia, Veszprém, Hungary; 168 Department of Biology and Inland Waters Protection, University of Belgrade-Institute for Multidisciplinary Research, Belgrade, Serbia; 169 Department of Ecology, University of Innsbruck, Innsbruck, Austria; 170 Department of Biological Sciences, Rensselaer Polytechnic Institute, Troy, USA; 171 Darrin Fresh Water Institute, Rensselaer Polytechnic Institute, Bolton Landing, USA; 172 National Institute for Marine Research and Development “Grigore Antipa”, Constanta, Romania; 173 HUN-REN Balaton Limnological Research Institute, Tihany, Hungary; 174 Estonian Maritime Academy, Tallinn University of Technology, Tallinn, Estonia; 175 Department of Aquatic Ecology, Netherlands Institute of Ecology, Wageningen, The Netherlands; 176 Fisheries and Aquatic Ecosystems, Agri-Food and Biosciences Institute, Belfast, UK; 177 Cawthron Institute, Nelson, Aotearoa New Zealand; 178 Centre for Nature Positive Solutions, Biology Discipline, RMIT University, Melbourne, Australia; 179 Department of Chemical Engineering, Cyprus University of Technology, Limassol, Cyprus; 180 Servicio Departamental de Salud La Paz, La Paz, Bolivia; 181 Ecosystems and Environment Research Program, Faculty of Biological and Environmental Sciences, University of Helsinki, Helsinki, Finland; 182 Laboratory of Ecophysiology and Molecular Evolution, Biodiversity, Brazilian National Institute for Research of the Amazon, Manaus, Brazil; 183 Surface Geosciences Department, Earth Sciences New Zealand, Lower Hutt, Aotearoa New Zealand; 184 Department of Aquatic Resources, Faculty of Natural Resources and Agricultural Sciences, Swedish University of Agricultural Sciences, Stockholm, Sweden; 185 Chair of Aquaculture, Institute of Veterinary Medicine and Animal Sciences, Estonian University of Life Sciences, Tartu, Estonia; 186 Institute of Computer Science, University of Tartu, Tartu, Estonia; 187 Cyprus University of Technology, Limassol, Cyprus; 188 Department of Environmental and Biological Sciences, University of Eastern Finland, Joensuu, Finland; 189 Tvärminne Zoological Station, University of Helsinki, Hanko, Finland; 190 Freshwater and Marine Ecology, Institute for Biodiversity and Ecosystem Dynamics, University of Amsterdam, Amsterdam, The Netherlands; 191 Institute for Environmental Sciences, University of Kaiserslautern-Landau (RPTU), Landau, Germany; 192 Department of Estuarine and Ocean Sciences, School for Marine Science and Technology, University of Massachusetts Dartmouth, New Bedford, USA; 193 Science, Engineering and Built Environment, Centre for Marine Science, Deakin University, Burwood, Australia; 194 Biodesign Lab, Delft University of Technology, Delft, The Netherlands; 195 Biology, College of Arts and Sciences, East Stroudsburg University, East Stroudsburg, USA; 196 Department of Environmental Management, Waterways, Lincoln University, Lincoln, New Zealand; 197 Chair of Urban Water Systems Engineering, Technical University of Munich, Garching, Germany; 198 Department of Biological Sciences, University of Santo Tomas, Manila, Philippines

## Abstract

Advances in high-throughput DNA sequencing technologies have accelerated research on aquatic microorganisms, their diversity, and ecological functions. This has also significantly advanced research on fungi that shape aquatic ecosystems and food webs. Here, we present the FunAqua dataset, which describes global fungal biodiversity in aquatic ecosystems based on eukaryotic long-read metabarcoding data from 1,076 sediment and 1,299 filtered water samples collected across 1,452 sampling sites in 87 countries. We targeted the internal transcribed spacer (ITS) region. In total, 50,152 fungal OTUs were recorded. The sequencing data is accompanied by comprehensive sample metadata, including physicochemical data of water and sediments. The FunAqua dataset provides a new resource for analysing and better understanding the biodiversity of fungi in different types of aquatic habitats across biogeographical regions. The dataset described in this data paper will be periodically updated with additional metabarcoding data.

## Background & Summary

Fungi are ubiquitous in aquatic environments, thriving in both marine and freshwater habitats^1^. They are important contributors to the functioning of aquatic ecosystems, primarily as parasites, decomposers, or mutualists. Parasitic fungi can regulate toxic algal blooms and, in the process, produce zoospores edible to zooplankton, promoting trophic transfer via the mycoloop^2^. In freshwater streams, the decomposition of plant material, including leaves, twigs and pollen, is primarily driven by fungi^3^. Lastly, previous research indicates that these fungi establish various types of mutualistic relationships with different organisms. For example, arbuscular mycorrhizal fungi colonise the roots^4^ of aquatic plants. There are also semiaquatic lichens and corresponding lichenicolous^5^ fungi.

Although fungi in aquatic ecosystems play diverse and ecologically significant roles, they are understudied and their global diversity is poorly documented compared to other aquatic microorganisms and terrestrial fungi^6^. Furthermore, metabarcoding has revealed an abundance of aquatic dark matter fungi, which lack ecological, taxonomic, and morphological descriptions, highlighting the need for further research^7^. A more comprehensive understanding of aquatic fungal communities is essential for defining their ecological roles, including broader impacts that they have on ecosystem functioning as well as the environmental factors shaping their diversity and distribution.

Advances in high-throughput sequencing technologies, particularly metabarcoding, have revealed an unexpectedly high aquatic fungal diversity that has remained undetected by microscopy and cultivation-based methods^8^. However, current knowledge about global aquatic fungal biodiversity and distribution still remains limited, and fungal sequences isolated from aquatic samples are underrepresented in reference databases^9^. Two online resources, https://marinefungi.org^10^ and https://freshwaterfungi.org^11^ document and classify marine and freshwater fungi, respectively. Additionally, a database named ParAquaSeq contains rRNA sequences of zoosporic parasites, including zoosporic parasitic fungi^12^. Nevertheless, comprehensive, publicly available metabarcoding datasets with a global scope and uniform methodology are still lacking for the vast majority of fungi in aquatic ecosystems.

**Fig. 1:**
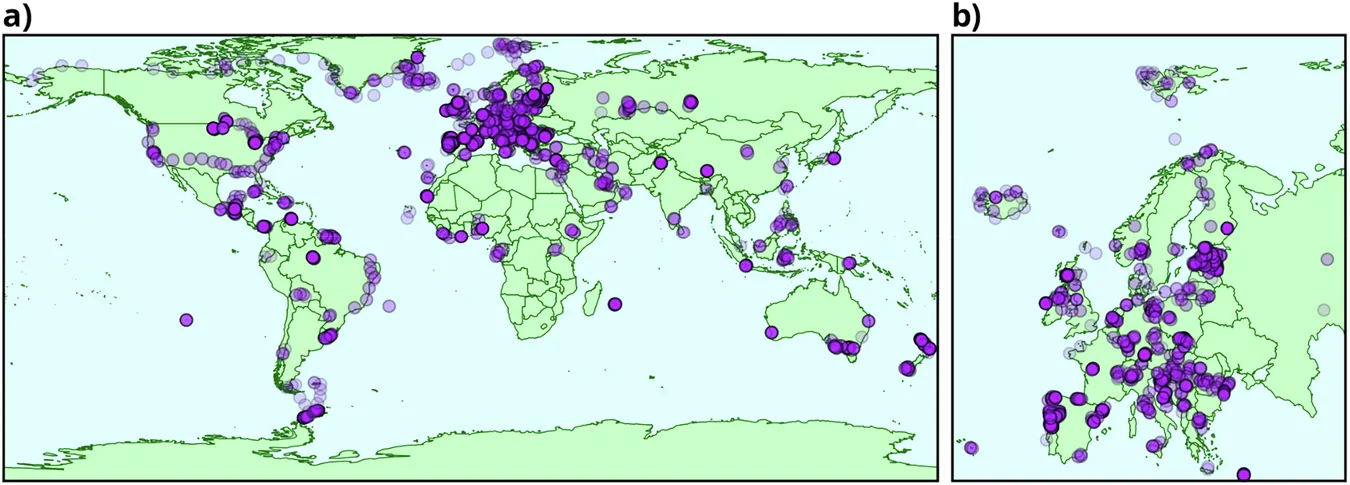
(**a**) Global distribution of sampling sites for the FunAqua dataset. (**b**) Zoomed in view of the European sampling plots.

Assembling datasets from published sequencing data is challenging due to variations in methodology and insufficient metadata^13^. These challenges can significantly impact research outcomes and limit the ability to conduct comparative analyses. To advance our understanding of the diversity of fungi in aquatic ecosystems, we initiated the collaborative project “FunAqua”. This project aims to explore and document the diversity of fungi in aquatic ecosystems and the environmental factors that shape their biodiversity, contributing to a deeper understanding of their ecological roles and distribution patterns in both freshwater and marine ecosystems. Here we provide and describe the FunAqua dataset which comprises eukaryotic sequences obtained from 1,299 filtered water and 1,076 sediment samples collected from 1,452 sampling sites in 87 countries across all continents (Fig. 1a,b). Metabarcoding was based on amplification and sequencing of the entire internal transcribed spacers (ITS) region^14^. The ITS region is a universal fungal barcode^15^ and has the most extensive reference database coverage among the rRNA operon barcodes^16^. In addition to the raw and processed sequencing data, the FunAqua dataset is accompanied by detailed environmental metadata, providing valuable context for interpreting community composition and ecological patterns.

## Methods

### Organization

The FunAqua project was established as a global, collaborative, voluntary initiative to advance the understanding of fungal biodiversity across diverse freshwater and marine ecosystems in countries around the world (Fig. 1). To ensure the robustness and comparability of data across a range of different aquatic environments, protocols were developed for all key steps, including sample collection, filtration, preservation and transportation of water and sediment samples. Two protocol versions were provided to participants: (1) basic protocols for general sample collection, suitable for a wide range of interested users; and (2) advanced protocols for researchers with access to specific equipment to collect additional environmental data (e.g., depth, water temperature, pH, and dissolved oxygen). Sampling and sample processing protocols are published and described in detail in Panksep *et al*. (2023^17^, 2025^18^).

While some variation was inevitable due to local and environmental constraints (e.g., the type of filters and the preservatives used), all participating teams adhered to the core guidelines to ensure data consistency. Any deviations were transparently documented in the metadata report sheet (Supplementary Item 1) to support accurate interpretation and data standardisation.

After sampling, collaborators sent their samples to a central laboratory at the Estonian University of Life Sciences (EMU), where all samples were registered and stored until further processing. All molecular analyses, including DNA extraction, PCR amplification, and library preparation were conducted at the Mycology and Microbiology Center, University of Tartu (UT), ensuring centralised quality control and consistency across laboratory workflows.

### Sampling

Between March 2018 and September 2024, a total of 1,126 sediment samples, 1,351 filtered water samples and 558 unfiltered water samples, collected by the consortium, were analysed. Filtered water samples and sediment samples were collected for metabarcoding, and unfiltered water for chemical analyses. Of the processed samples, 1,076 sediment and 1,299 filtered water samples were successfully sequenced and are included in the current version of the dataset. Metabarcoding data from the remaining samples will be incorporated with future dataset updates.

Water for eDNA metabarcoding was filtered using 12 different types of filters, including Sterivex^TM^ units (n = 754; Millipore, USA), cellulose-based (n = 313), glass-filled polytetrafluoroethylene (n = 2), polyvinylidene fluoride (n = 12), nylon (n = 11), polycarbonate (n = 69), polyethylene (n = 8) and polyethersulfone (n = 49) filters. The filters used for 80 of the samples were not specified by the sample providers. The majority of the filters (n = 1,285) had a pore size of 0.2–0.22 µm as predefined in the protocol. A small subset (n = 14) of samples were filtered with filters that had a pore size of 0.1 µm. Water collected for filtration was filtered immediately after sampling on-site or within the day of sample collection. Both sediment and filtered water samples were immediately preserved in ethanol and stored at - 20 °C until further processing. In locations where maintaining a cold chain for sample preservation and transportation was not feasible, samples (n = 397) were preserved using Longmire’s buffer^19^, which is widely employed in eDNA workflows for its ability to preserve nucleic acids at ambient temperature and inhibit nuclease activity^20^. Samples preserved in ethanol were shipped frozen on dry ice or ice packs in insulated shipping containers for subsequent molecular and chemical analyses.

### Molecular analysis

#### DNA extraction

Sediment samples were placed in envelopes and dried overnight in an air-drying cabinet at 35 °C. The dried material was then transferred to 2-mL tubes and homogenised using an MM 200 Mixer Mill (RETSCH, Germany) with three 2-mm metal beads at 30 Hz for 10 mins. DNA was extracted from 0.2 g of homogenised sediment samples using the MagAttract PowerSoil DNA Kit (Qiagen, Germany) and a KingFisher™ Flex machine (Thermo Fisher Scientific), according to the manufacturer’s protocol. Additional DNA extractions were conducted on sediment samples (n = 367) that failed to amplify during the initial PCR run. The re-extractions followed the same protocol without modifications, as the aim was to verify that the absence or the low number of sequences was not due to issues in the original DNA extraction process.

DNA from filtered water samples was extracted using the NucleoMag^®^ DNA/RNA Water kit (Macherey-Nagel, Germany) with a modified protocol performed on the KingFisher™ Flex machine. Additional steps were incorporated to extract the DNA from the preservation buffer in addition to the filter. The buffer was transferred from the filters to 2-mL tubes and centrifuged for 5 mins at 11,000 g. The supernatant was removed, and the remaining pellet was dried at 56 °C. Sterivex^TM^ filter units were dried at 56 °C to evaporate any remaining buffer. Other types of filters were removed from their tubes in a laminar flow hood, air-dried on sterile petri dishes, and transferred into 5-mL type A bead tubes. Then, 900 µL of C1 and MWA1 lysis solutions were added to the dried pellets from Sterivex^TM^ tubes and non-Sterivex^TM^ tubes, respectively. The pellets were resuspended and mixed with the lysis solution by pipetting 10 times, and the solution was transferred back onto the original respective filters. If no buffer remained in the Sterivex units, buffer C1 was added directly to the filter. The rest of the extraction process largely followed the manufacturer’s instructions with two exceptions. The incubation step at 70 °C was extended from 5 mins to 10 mins to enhance the lysis of fungal cell walls. Secondly, the binding, washing and elution steps were performed according to the manufacturer’s protocol, but on a KingFisher™ Flex machine.

#### PCR amplification and preparation for sequencing

PCR amplification of the rRNA operon full ITS region (average amplicon length 866 bp) was performed using the ITS9mun/ITS4ngsuni primer pair^14^. Both primers were indexed with one of 115 12-base indices that differed from each other by at least four nucleotides^21^. The PCR reactions were carried out in two 25-µL replicates, which were pooled thereafter. The PCR reaction mix contained 5 µL of 5 × HOT FIREPol Master Mix (Solis BioDyne, Estonia), 0.5 µL of both reverse and forward primers (20 µM), 1 µL of template DNA, and 18 µL of ddH_2_O. Thermal cycling included initial denaturation at 95 °C (15 min), followed by 30 cycles of denaturation at 95 °C (30 seconds), primer annealing at 55 °C (30 seconds) and extension at 72 °C (1 min). The program ended with a final extension step at 72 °C (1 min). PCR products were visualised on a 1% agarose gel under UV illumination at 280 nm. Products with a weak or no visible band were reamplified using 33–38 cycles with an increased (2-3 µL) amount of DNA. If increasing the amount of template DNA did not yield visible bands on an agarose gel, the amount of template DNA was decreased to 0.5 µL to account for potential PCR inhibition due to co-extracted chemicals. Each PCR reaction included a negative control to check for cross-contamination and a positive control (UniSpike, an artificial DNA molecule) to ensure that any amplification failures were not due to systemic issues.

PCR products of various amplicons were pooled based on band intensity observed on agarose gels. Pooled libraries were purified using the FavorPrep^TM^ GEL/PCR Purification kit (FAVORGEN BIOTECH CORP, Taiwan) following a modified protocol. Specifically, pooled libraries were mixed with 3 volumes of FADF buffer (instead of the standard 5 volumes), and purification was performed in duplicates using two separate columns. Each column was eluted in 60 µL of the elution buffer, and the resulting eluates were combined to yield a final volume of 120 µL.

The pooled libraries were sent to the Norwegian Sequencing Centre (University of Oslo, Norway) for library preparation and sequencing. Library preparation and DNA sequencing were performed using the PacBio Sequel II System (Pacific Biosciences, Palo Alto, USA) as described previously^21^.

### Bioinformatics

Bioinformatic processing followed the open-source NextITS workflow v1.0.0^22^, orchestrated with Nextflow v25.04.6^23^ and containerised with Singularity^24^. Demultiplexing of PacBio HiFi reads (circular consensus sequences, CCS) was performed with LIMA v2.12.0 (Pacific Biosciences). For primer trimming, cutadapt v5.0^25^ was used with two errors allowed and a minimum overlap of 10 nucleotides for both forward and reverse primers. Reads lacking both primer sites in the correct orientations were discarded. Sequences shorter than 200 nucleotides were removed. During quality filtering, we excluded reads with more than 0.6 expected errors per 100 bp (computed from Phred quality scores on a per-read, length-normalized basis), homopolymers >25 nucleotides, and >4 ambiguous bases. Full-length ITS regions were retrieved with ITSx v1.1.3^26^. Chimeric sequences were removed using a two-step scheme by first applying *de novo* (with a maximum chimera score of 0.6)^27^ and then reference-based filtering (using the EUKARYOME v1.9.3 database)^16^ algorithms within VSEARCH v2.29.4^28^.

Tag-jump (index cross-talk) removal was performed using the UNCROSS2 algorithm^29^. Dereplicated, abundance-sorted reads were denoised using UNOISE3^30^, with parameters alpha = 6 and minsize = 1, and clustered into OTUs at 98% pairwise sequence similarity using VSEARCH v2.29.4^28^. Taxonomic annotation of representative sequences was performed using BLASTn v2.16.0+, where EUKARYOME v1.9.3^16^ served as a reference database.

### Chemical analysis

#### Unfiltered water samples

Chemical analysis was conducted on a subset of 558 unfiltered water samples, which were sent frozen to EMU. The analyses included measurements of electrical conductivity, pH, total phosphorus (TP), and total nitrogen (TN). Conductivity and pH were measured with XS COND 8 (XS Instruments, Italy) and inoLab^®^ pH 7110 (WTW) benchtop meters (Xylem Analytics, Germany), respectively. TP concentrations were determined using the molybdenum blue spectrophotometric method^31^ after oxidation to phosphate (PO_4_^3−^) with potassium persulfate (K_2_S_2_O_8_). This method conforms to the international standard for the determination of phosphorus in ground, surface, and waste waters^32^. TN concentrations were determined using the ultraviolet spectrophotometric screening method^33^ after oxidation to nitrate (NO_3_^−^) with K_2_S_2_O_8_. This method conforms to the international standard for determination of nitrogen using oxidative digestion with peroxodisulfate^34^.

#### Sediment samples

Nutrient analysis was performed for a subset of 463 sediment samples collected from 382 sampling sites. Dried sediment samples (0.25 g) were transferred into 50-mL PTFM vessels and dissolved in HNO_3,_ using the Multiwave Pro (Anton Paar, Austria) system equipped with a 24HVT50 rotor (Anton Paar). The digestion step followed the EPA 3051 A standard method^35^. The Montana soil sample NIST^®^ 2711a (National Institute of Standards and Technology, USA) was included in each digestion sequence as a quality control.

Digests were analysed using the 4210 MP-AES (Agilent, USA) instrument equipped with CETAC^TM^ U5000AT +Ultrasonic Nebulizer (ThermoFisher Scientific) to enhance analytical signal strength. Calibration ranges of elements and analytical wavelengths for each element are provided in the dataset (see the Data Records chapter). Sediment samples from batches 1, 2 and 4 were diluted 10-fold with sterile deionized water prior to the analysis. Samples that were fully dissolved during digestion were analysed separately in batch 3 after a 30-fold dilution due to the high element concentrations in the digest. NIST^®^ 2711a control samples were also diluted 30-fold for consistency.

A secondary calibration standard was run for every 15–20 samples throughout the analytical sequence. A third-order polynomial fit of the secondary calibration data was used to correct for a time-dependent instrumental drift. For quality control, a 10-fold dilution of NIST^®^ 1643 F (Trace Elements in Natural Water, National Institute of Standards and Technology), spiked with 1 mg/L phosphorus using a single-element P standard, was included in each analytical run alongside the secondary calibration standards. Average recoveries from the NISTNIST^®^ 1643 F and NIST^®^ 2711a are presented in the dataset (see the Data Records chapter). Duplicate measurements of the same sample aliquots within a single analytical run showed <5% deviation in elemental concentrations.

## Data Records

The dataset is accessible via Zenodo^36^. The dataset is organized into 8 parts (Table 1): the metadata table, the water chemistry dataset, the sediment elemental analysis data, and the sediment elemental analysis reference data; the fungal and non-fungal OTU tables; and the fungal and non-fungal taxonomy tables. A data dictionary file describing all fields, including units of measurement wherever applicable, is also included in the dataset.

**Table 1:** FunAqua dataset composition.

File name	File function	Description	File format
Funaqua_metadata.tsv	Sample data	Metadata corresponding to each sample	Tab-delimited text file (.tsv)
FunAqua_fungi_otu.csv	OTU abundance data	Table containing sequence counts of each fungal OTU in each sample	Comma-separated text file (.csv)
FunAqua_euk_otu.csv	OTU abundance data	Table containing sequence counts of each non-fungal OTU in each sample	Comma-separated text file (.csv)
FunAqua_fungi_tax.csv	Taxonomy table	Table with taxonomic classification data for fungal OTUs	Comma-separated text file (.csv)
FunAqua_euk_tax.csv	Taxonomy table	Table with taxonomic classification data for non-fungal OTUs	Comma-separated text file (.csv)
FunAqua_Water chemistry_Data.csv	Water chemistry data	Table containing results of water chemistry analyses	Comma-separated text file (.tsv)
FunAqua_sediment_chemistry.csv	Sediment elemental analysis data	Table containing results of sediment elemental analyses	Comma-separated text file (.csv)
FunAqua_sediment_chemistry_references.csv	Sediment elemental analysis reference data	Includes analyses of calibration ranges for reference data	Comma-separated text file (.csv)

Raw sequencing data can be obtained at the European Nucleotide Archive under bioproject PRJEB104083^37^ (biosamples SAMEA120633854 - SAMEA120634929; SAMEA120637523 - SAMEA120638821) in FASTQ format. The demultiplexed sequences have been trimmed of primers and indices. OTU representative sequences (FASTA files), OTU tables and associated taxonomic assignments will also be published through the PlutoF platform and made available through GBIF as DNA-derived biodiversity occurrence data.

The FunAqua dataset is published under Creative Commons BY 4.0 license and is freely available to use.

### Metadata

To ensure consistency in data reporting and facilitate downstream analyses, a unified metadata template was developed, which standardised the recording of sample-specific information, environmental variables, and any protocol deviations, enabling the integration of data into a comprehensive, harmonised dataset.

The metadata and biological data structure are detailed in the following tables: Table 2 presents the fields used for FunAqua sample metadata. Table 3 defines the fields of the FunAqua OTU abundance data, where the OTU field serves as the primary key variable linked to the taxonomic table, and the remaining fields represent unique sample identifiers containing their respective OTU abundance values. Table 4 describes the fields of the FunAqua taxonomic classification data, providing the hierarchical lineage for each OTU from kingdom to species level, using the OTU query ID as the key variable for integration with the abundance data.

**Table 2:** Fields for the FunAqua metadata.

Column name	Dataset	Description
sample_code	General metadata	Identifier for samples
plot_name	General metadata	Name or identifier of the sampling plot or site
sample_tue	General metadata	Accession IDs assigned by the University of Tartu repository
sample_source	General metadata	Environmental matrix from which the sample was collected
sample_source_description	General metadata	Additional details about the sample source
sampling_point_depth	General metadata	Depth at which the sample was collected
date	General metadata	Date of sample collection in ISO format
country	General metadata	Country of sample collection
continent	General metadata	Continent of sample collection
location_name	General metadata	Specific location name of the sample collection
lat	General metadata	Latitudinal coordinate of the sampling location
long	General metadata	Longitudinal coordinate of the sampling location
biome	General metadata	Ecological biome classification of the sampling site (e.g., freshwater lake, river, wetland)
elevation	General metadata	Elevation of the waterbody surface during observation relative to the sea level
amount_filtered	General metadata	Volume of material filtered during sample processing
filter_type	General metadata	Type of filter used for sample processing
filter_pore_size	General metadata	The size of the filter pores
plot_name	Water chemistry data	Name or identifier of the sampling plot or site
Conductivity	Water chemistry data	Electrical conductivity of the sample
pH	Water chemistry data	Measure of the acidity or alkalinity of the sample
TP	Water chemistry data	Total phosphorus concentration in the sample
TN	Water chemistry data	Total nitrogen concentration in the sample
plot_name	Sediment chemistry data	Name or identifier of the sampling plot or site
sample_weight	Sediment chemistry data	Mass of the sediment sample used for analysis
P_mg_per_kg	Sediment chemistry data	Phosphorus concentration
P_SD	Sediment chemistry data	Standard deviation of phosphorus concentration
Fe_mg_per_kg	Sediment chemistry data	Iron concentration
Fe_SD	Sediment chemistry data	Standard deviation of iron concentration
Mg_mg_per_kg	Sediment chemistry data	Magnesium concentration
Mg_SD	Sediment chemistry data	Standard deviation of magnesium concentration
Na_mg_per_kg	Sediment chemistry data	Sodium concentration
Na_SD	Sediment chemistry data	Standard deviation of sodium concentration
Ca_mg_per_kg	Sediment chemistry data	Calcium concentration
Ca_SD	Sediment chemistry data	Standard deviation of calcium concentration
K_mg_per_kg	Sediment chemistry data	Potassium concentration
K_SD	Sediment chemistry data	Standard deviation of potassium concentration
batch	Sediment chemistry data	Name of the measurement batch
sample	Sediment chemistry data	Name of the reference material used
P_mg_per_kg	Sediment chemistry data	Phosphorus concentration
P_SD	Sediment chemistry data	Standard deviation of phosphorus concentration
Fe_mg_per_kg	Sediment chemistry reference data	Iron concentration
Fe_SD	Sediment chemistry reference data	Standard deviation of iron concentration
Mg_mg_per_kg	Sediment chemistry reference data	Magnesium concentration
Mg_SD	Sediment chemistry reference data	Standard deviation of magnesium concentration
Na_mg_per_kg	Sediment chemistry reference data	Sodium concentration
Na_SD	Sediment chemistry reference data	Standard deviation of sodium concentration
Ca_mg_per_kg	Sediment chemistry reference data	Calcium concentration
Ca_SD	Sediment chemistry reference data	Standard deviation of calcium concentration
K_mg_per_kg	Sediment chemistry reference data	Potassium concentration
K_SD	Sediment chemistry reference data	Standard deviation of potassium concentration
Batch	Sediment chemistry reference data	Name of the measurement batch

**Table 3:** The fields of FunAqua OTU abundance data.

Field	Description
OTU	OTU query ID. Key variable for this table and the taxonomic table (see Table 4).
Remaining fields	Field names are unique sample identifiers. Values are OTU abundance numbers of the OTU corresponding to the row.

**Table 4:** The fields of FunAqua taxonomic classification data.

Field	Description
OTU	OTU query ID. Key variable for the OTU abundance table
Kingdom	Assigned kingdom
Phylum	Assigned phylum
Class	Assigned class
Order	Assigned order
Family	Assigned family
Genus	Assigned genus
Species	Assigned species

We used the terminology and standards of Darwin Core^38^ and MIMARKS^39^ to facilitate machine readability and communication.

Sample collection sites were assigned alphanumeric IDs in the format “W” followed by 4 digits (e.g., W0001). Samples collected from each site were given a suffix “_w1” or “_s1”, indicating type as filtered water or sediment samples, respectively. The samples for which metadata had already been uploaded to the PlutoF^40^ cloud data manager by the sample collectors lack ID’s following the former system and use only the accession ID’s assigned by the repository of the University of Tartu (acronym TUE, with 6-digit accession numbers). All other physical samples and their respective DNA samples are also deposited in TUE.

We categorised sampling sites into 10 different biomes (Fig. 3). These categories were generalised for uniformity purposes. Aquatic habitats, which could not be categorised under the 10 biomes, are labelled as “aquatic: other”.

## Technical Validation

### Location data verification

Latitude and longitude records for each waterbody were manually verified using Google Maps (satellite and map views). A location was marked as verified if it met one of the following criteria:

1. corresponded to a waterbody with a matching *plot name*;
2. was in or close to a waterbody with no name or a different name, but the provided location name matched a nearby city or region
3. was near an unnamed waterbody in the correct country, with no other waterbodies nearby.

Locations that did not meet any of these criteria were considered unverified. In such cases, data collectors were contacted by the FunAqua coordinator to confirm or correct the coordinates (latitude and longitude in decimal degrees; WGS84).

### Sampling coverage

**Fig. 2:**
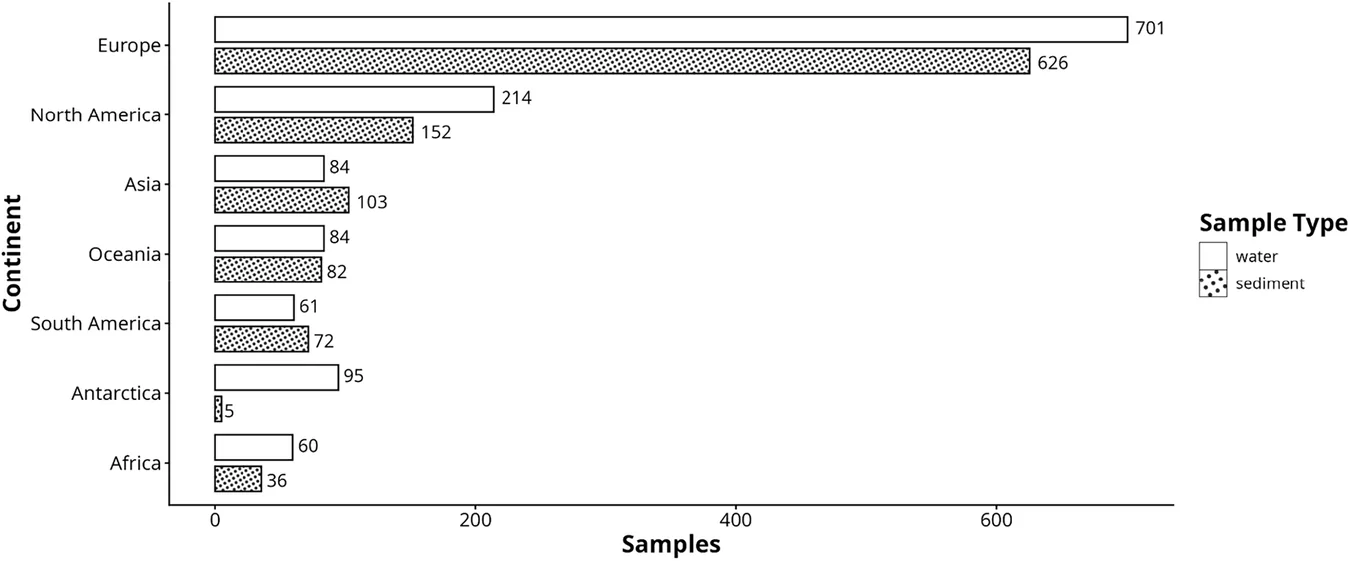
Number of sediment and filtered water samples collected from each continent in the FunAqua dataset.

Of the 1,452 sampling sites included in the dataset, 63% (920 sites) had both sediment and filtered water samples. The sampling sites were predominantly located in Europe, and the 1,327 European samples accounted for 56% of all samples (Fig. 2). Samples from North America accounted for 15% of the total samples and this was the continent that supplied the second highest number of samples.

**Fig. 3:**
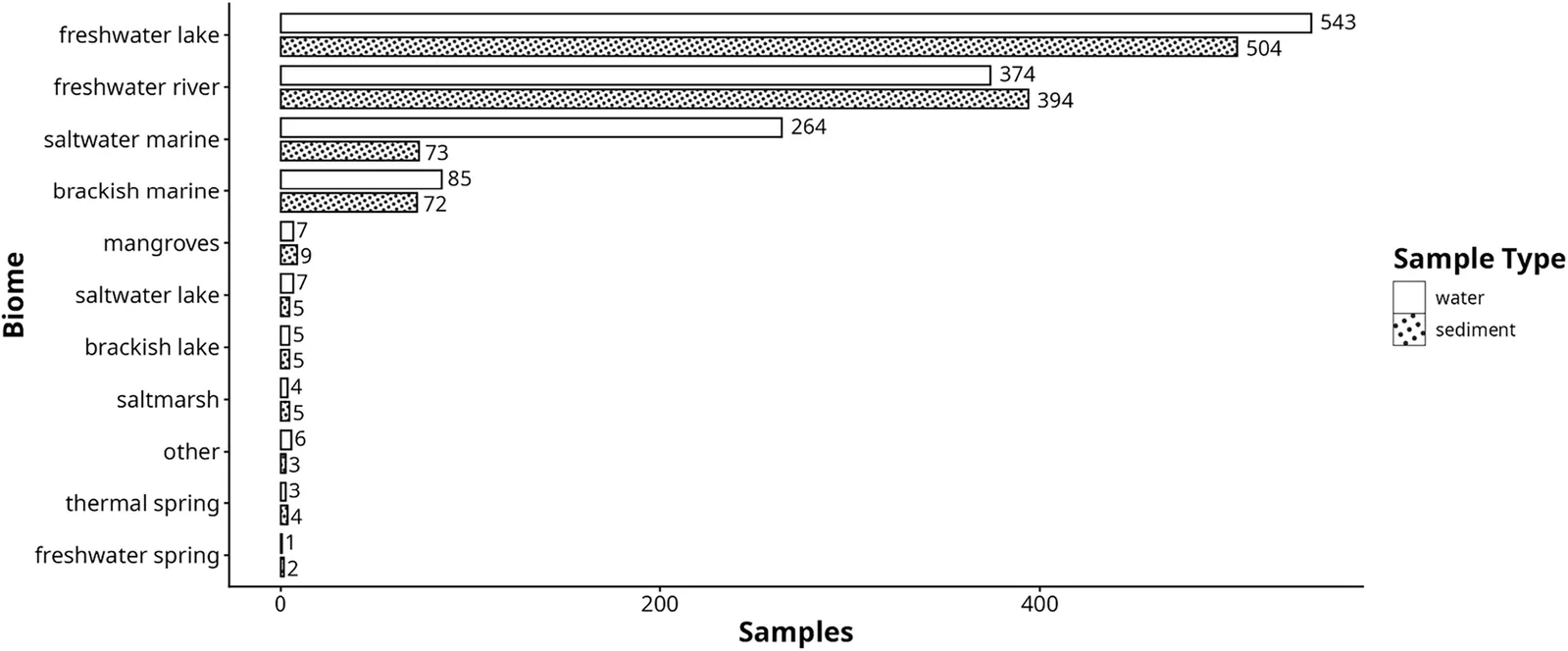
Number of sediment and filtered water samples collected from each biome in the FunAqua dataset.

The largest proportion of samples were collected from freshwater lakes (44%; 1047 samples), followed by freshwater rivers (32%; 768), saltwater marine (14%; 337) and brackish marine (7%; 157) environments (Fig. 3). Mangroves (16 samples), saltwater lakes (12), brackish lakes (10), salt marshes (9), thermal springs (7) and freshwater springs (3) were much less represented in sampling (Fig. 3).

**Fig. 4:**
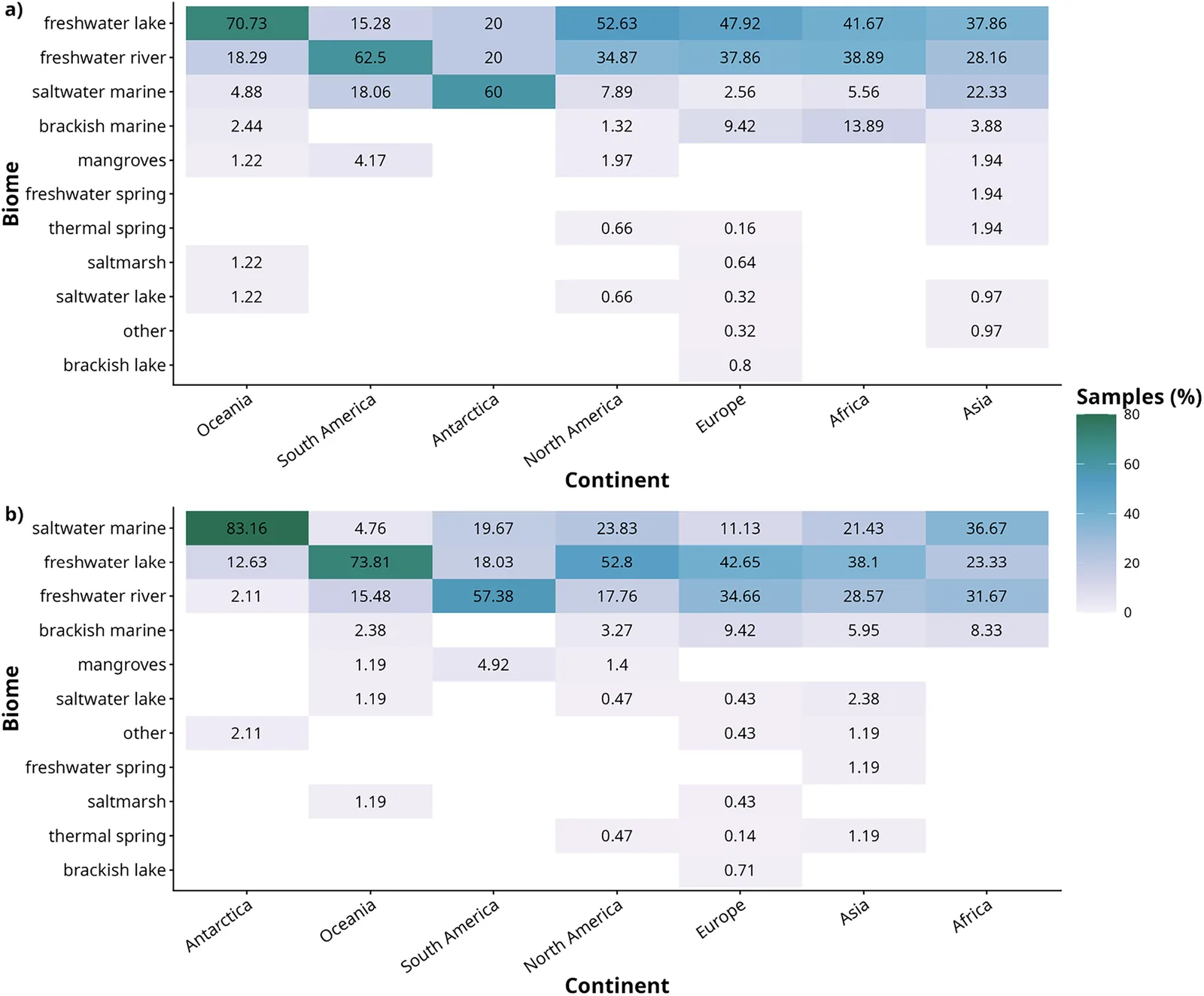
Heat map charts depicting the proportion (%) of samples collected from each biome on each continent in the FunAqua dataset. (**a**) Proportions of sediment samples from each biome on each continent. (**b**) Proportions of filtered water samples from each biome on each continent.

Freshwater lake, freshwater river, and saltwater marine biomes were sampled on all continents. Brackish marine samples were absent from the Antarctic and South American sample sets. Sampling campaigns conducted in Europe and Asia covered the greatest number of biome types (8 biomes each), followed by North America (7) (Fig. 4). Samples from Antarctica were predominantly of saltwater marine origin, comprising 60% of sediment samples and 83% of filtered water samples.

### Contamination control

Each sequencing library and PCR reaction included both a positive and a negative control. Fungal OTUs were detected in 19 out of 28 negative controls. The majority of these OTUs were either low quality or showed signs of contamination, likely originating from the positive control. High-quality fungal OTUs detected in the negative controls were more abundant and common in other samples within each sequencing library, indicating minor cross-contamination. There were no signs of library-wide contamination, as there were no OTUs which were present in the majority of samples in a library and in the negative control. OTUs deemed to be caused by contamination were removed from the dataset.

### Bioinformatic validation

Amplicon integrity and marker correctness were validated through stringent sequence- and structure-based filtering. Only reads containing the complete set of expected index combinations and both primers in correct orientation were retained. Sequences with missing primers, inverted primer orientations, or multiple primer occurrences were discarded to remove non-specific products and reads misassembled during the generation of circular consensus PacBio sequences. To confirm that reads represented genuine full-length ITS amplicons, we processed all sequences with ITSx, requiring detection of rRNA regions (SSU, ITS1, 5.8S, ITS2, and LSU) in the correct order without overlap. Using the ITSx HMM profiles, we further required that all detected subregions within a sequence matched the same major eukaryotic lineage, enabling removal of cross-lineage chimeras and ensuring that only structurally consistent ITS sequences were retained for downstream analysis.

To minimise spurious diversity, we applied per-sample homopolymer correction to reduce systematic indel errors characteristic of long-read ITS sequencing, followed by global denoising with the UNOISE algorithm to collapse residual sequencing noise and artefactual sequence variants. Prior to clustering, reads were sorted jointly by abundance and average Phred score, ensuring that high-quality, well-supported sequences served as candidate OTU representatives.

Sequence processing followed the open-source NextITS workflow, orchestrated with Nextflow and containerised using Singularity, which ensures the full reproducibility of software versions and parameters across different computing environments.

### Taxonomic validation

To ensure the reliability of taxonomic assignments, we applied a multi-step quality control procedure. First, all OTUs were assigned taxonomy using BLASTn against the EUKARYOME reference database, which currently provides the most comprehensive coverage of aquatic fungal lineages as well as other eukaryotes commonly misidentified as fungi (e.g., Amoebozoa, Nucleariae, Choanoflagellata). Assignments were retained only when they met minimum thresholds for sequence identity (65%), alignment coverage (40%), and E-value (e-50). OTUs failing these criteria were assigned to the last reliable taxonomic rank or marked as unclassified at lower ranks.

Second, we manually inspected taxonomic assignments for known problematic groups, including environmental clades with uncertain phylogenetic placement and taxa prone to misidentification due to incomplete reference sequences. Conflicting assignments (where multiple high-scoring BLAST hits suggested different taxa) were resolved by examining the consensus across top hits and, where necessary, truncating assignments to higher taxonomic ranks to avoid overclassification. Reliable taxonomic ranks were determined using e-value and sequence similarity thresholds for species- to phylum-level assignments following Tedersoo *et al*. (2021^21^). Third, we flagged low-quality OTUs based on multiple criteria: (1) singletons and doubletons with PHRED quality scores <30; and (2) taxa with overall abundance <10 exhibiting three or more indel openings compared with the number of substitutions.

### Sequencing depth and taxonomic coverage

Overall, we analysed 1,126 sediment samples and 1,351 filtered water samples. No high-quality eukaryotic sequences could be detected in 50 sediment and 52 filtered water samples, likely due to the presence of PCR inhibitors. Additionally, the amount of DNA present on a filter depends on the volume of water filtered; thus, insufficient filtration volumes could yield suboptimal results. Samples with no remaining high-quality eukaryotic sequences were removed from the dataset and underwent further quality assessment processes. A subset (n = 367) of sediment samples were re-extracted and re-sequenced due to subpar results in the first round of sequencing. For these samples, a sequencing depth threshold of 350 reads was set. The copy meeting this threshold was retained in the dataset, while the other was discarded. If neither copy met this criterion, the OTU count data were merged. This process was conducted separately for non-fungal and fungal OTUs to ensure optimal coverage for both groups. The selection of the minimal sequencing depth threshold did not result in a significant loss of sequences or OTUs, as most discarded samples had fewer than 100 reads.

The total number of reads recovered was 13,064,293 (fungal: 2,888,694; non-fungal: 10,175,599). The OTU clustering resulted in a total of 496,061 eukaryotic OTUs, of which 132,417 (27%) represented fungi. After additional quality control using the methods described in taxonomic validation, 72% (358,253) of the detected OTUs were considered to be of low-quality. Of the remaining 137,808 high-quality OTUs (11,822,454 reads), the fungal kingdom comprised the largest subset of eukaryotic OTUs at 36.4% (50,152 OTUs; 2,065,407 reads), followed by Alveolata (16.6%; 22,936 OTUs; 3,618,896 reads) and Straminipila (13.6%; 18,744 OTUs; 1,256,493 reads). Kingdom-level classification could not be assigned to 650 OTUs (0.47%). The low-quality OTUs were excluded from the final dataset.

Sediments had a greater number of eukaryotic OTUs (90,865 OTUs; 6,048,432 reads) than water samples (74,110 OTUs; 5,774,022 reads), with 27,167 OTUs overlapping between the two sample types. Fungal OTUs were detected in 98% of the sediment samples (n = 1,056) and 93% of the filtered water samples (n = 1,212), from which 34,145 (1,335,319 reads) and 26,296 (730,088) fungal OTUs were recovered, respectively. Across all samples, fungi remained the most OTU-rich kingdom in both sediments (37.6% of OTUs) and water (35.5%).

The type of filter used for sample filtration had a significant effect on the number of log1p-transformed fungal sequences detected (Kruskal-Wallis chi-squared = 98.802, df = 11, p-value = 3.082e-16); however, these results should be interpreted with caution. Among the 66 pairwise comparisons of filter types, 20 showed clear differences in fungal sequence numbers (pairwise Wilcoxon test p < 0.05). Cellulose acetate filters differed significantly from nine other filter types in the number of detected log1p-transformed fungal sequences. However, the sample size for that specific type of filter was low (n = 7) and all these samples originated from the same general location.

The majority of OTUs in the dataset had a sequence count greater than one (82% of OTUs; 112,456 OTUs), while 25,352 OTUs (18%) were singletons. There was a slightly higher proportion of singletons among the fungal OTUs (21% of fungal OTUs; 10,714 OTUs) compared to other eukaryotes (17% of non-fungal OTUs; 14,638 OTUs). Based on the high-quality sequences, the overall median sequencing depth was 2,080 (IQR: 406–5,529.5). The median sequencing depth was greater in filtered water samples (2,330; IQR: 559.5–5072.5) than in sediments (1,658; 255.5–6345.5). For fungal sequences, the median sequencing depth was 125 (20–590), with sediments showing a higher median sequencing depth (196; 26–907.3) than filtered water samples (94; 15–381).

**Fig. 5:**
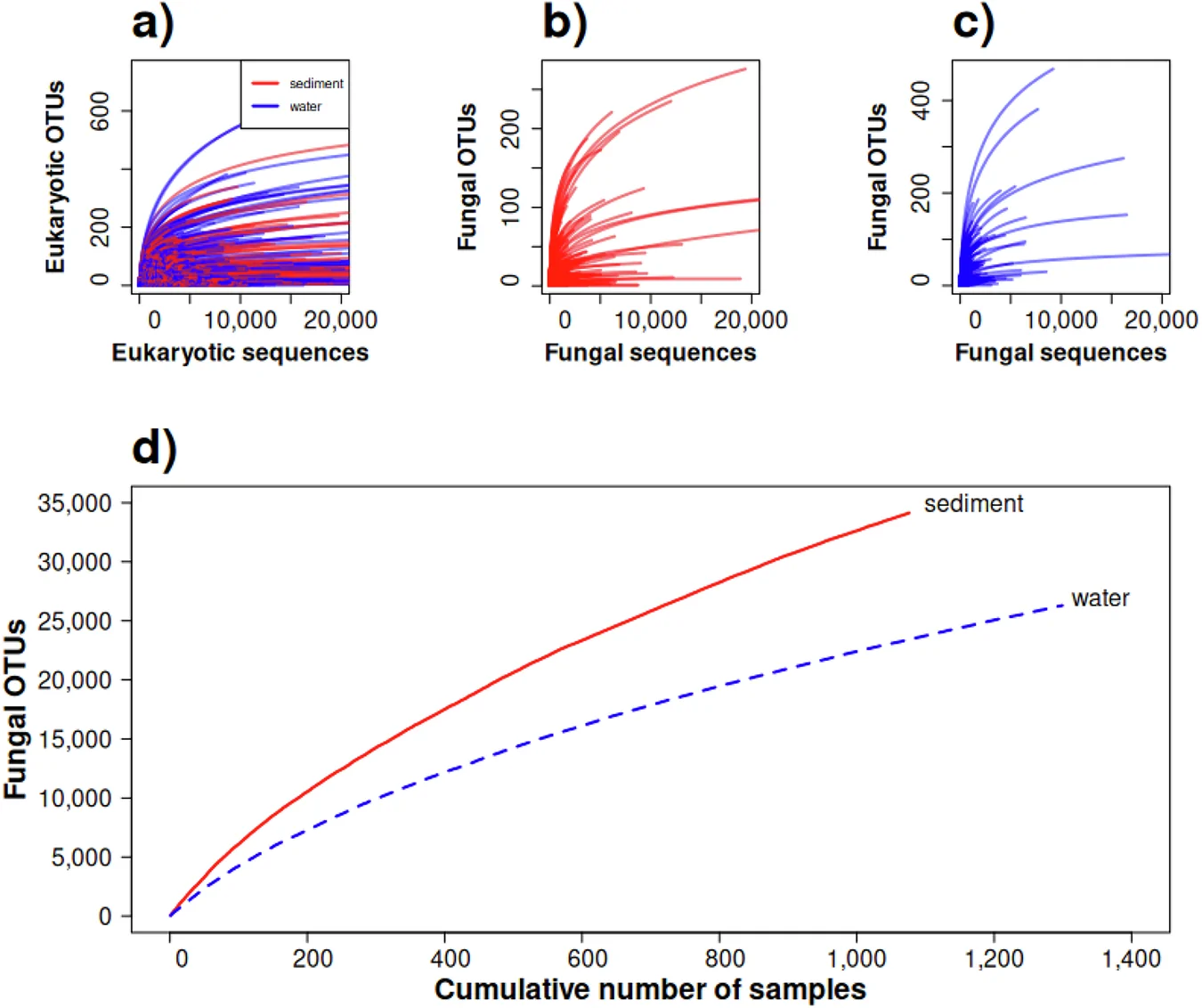
(**a**) Rarefaction curves of all eukaryotes in all samples in the FunAqua dataset. (**b**) Rarefaction curves of fungal OTUs in sediment samples. (**c**) Rarefaction curves of fungal OTUs in filtered water samples. d) OTU accumulation curve of fungal OTUs.

Rarefaction curves for most samples reached saturation or near-saturation when considering all high-quality eukaryotic OTUs (Fig. 5a). In contrast, rarefaction curves for fungal OTU accumulation suggested that sequencing depth was insufficient to reach saturation across all samples (Fig. 5b,c). The OTU accumulation curves (Fig. 5d) demonstrated a steady increase in the number of fungal OTUs detected across samples, with accumulation progressing more slowly in filtered water samples.

The lack of sufficient sequencing depth has been documented in previous studies that have used metabarcoding for the identification of fungi in aquatic ecosystems^41–43^. This is probably related to the small size of unpooled samples, and the widespread presence of air-borne spores from terrestrial fungal species, which can number in the millions^44^.

Based on the Good’s coverage index, 79% (n = 1,874) of samples achieved sufficient sequencing depth (C > 0.89), indicating that non-singleton OTUs accounted for at least 90% of the observed sequences. The median Good’s coverage index value across was 0.97 (IQR: 0.91–0.99). In contrast, 6% of samples (n = 141) had a high proportion of sequences belonging to singletons (≥50%), corresponding to low coverage values (C < 0.51). Analysis of fungal OTUs showed that only 57% of samples (n = 1,296) met the coverage threshold (C > 0.89), with a median index of 0.90 (IQR: 0.70–0.98). Notably, 20% of fungal samples (n = 454) exhibited ≥ 50% singleton OTUs (C < 0.51).

**Fig. 6:**
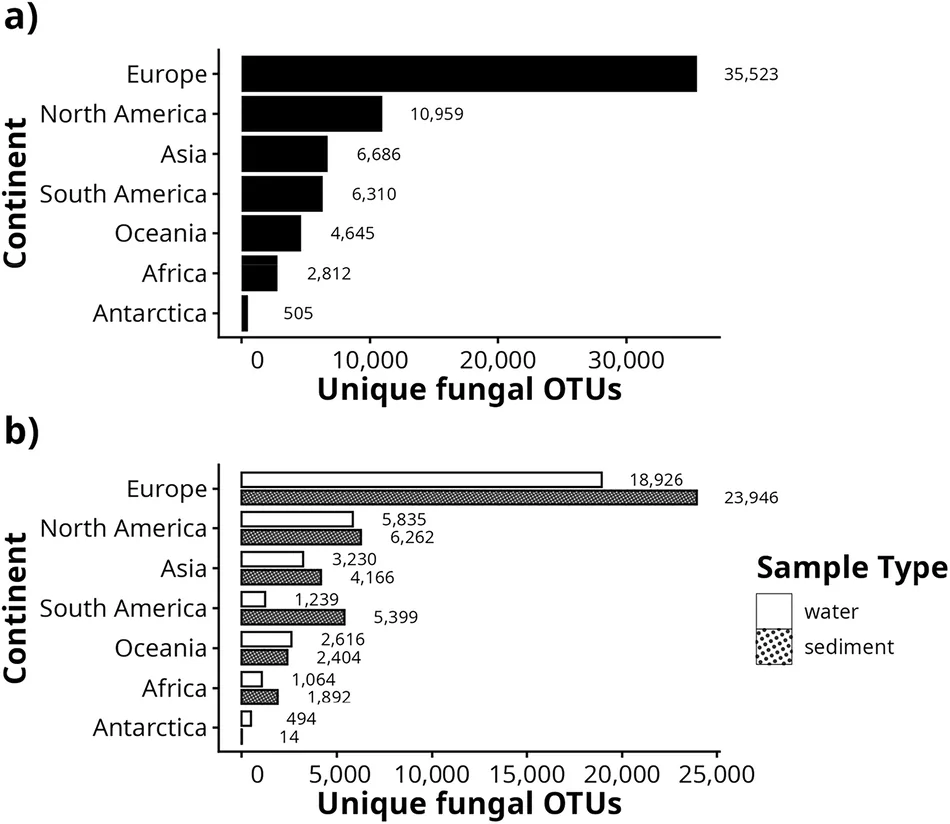
(**a**) number of unique fungal OTUs detected in all samples of each continent combined; (**b**) number of unique fungal OTUs detected in sediment and filtered water samples from each continent.

Continent-wise, fungal sequences were recovered from 1,295 European, 350 North American, 185 Asian, 162 Oceanian, 128 South American, 90 African, and 58 Antarctic samples. European samples had the highest number of high-quality fungal sequences (1,371,636 reads; 35,523 unique OTUs), followed by North America (310,230; 10,959) and Asia (145,444; 6,686) (Fig. 6a). Sediments had a higher number of detected OTUs on all continents, except for Oceania (water: 2,616 OTUs; sediments: 2,404) and Antarctica (494; 14) (Fig. 6b).

**Fig. 7:**
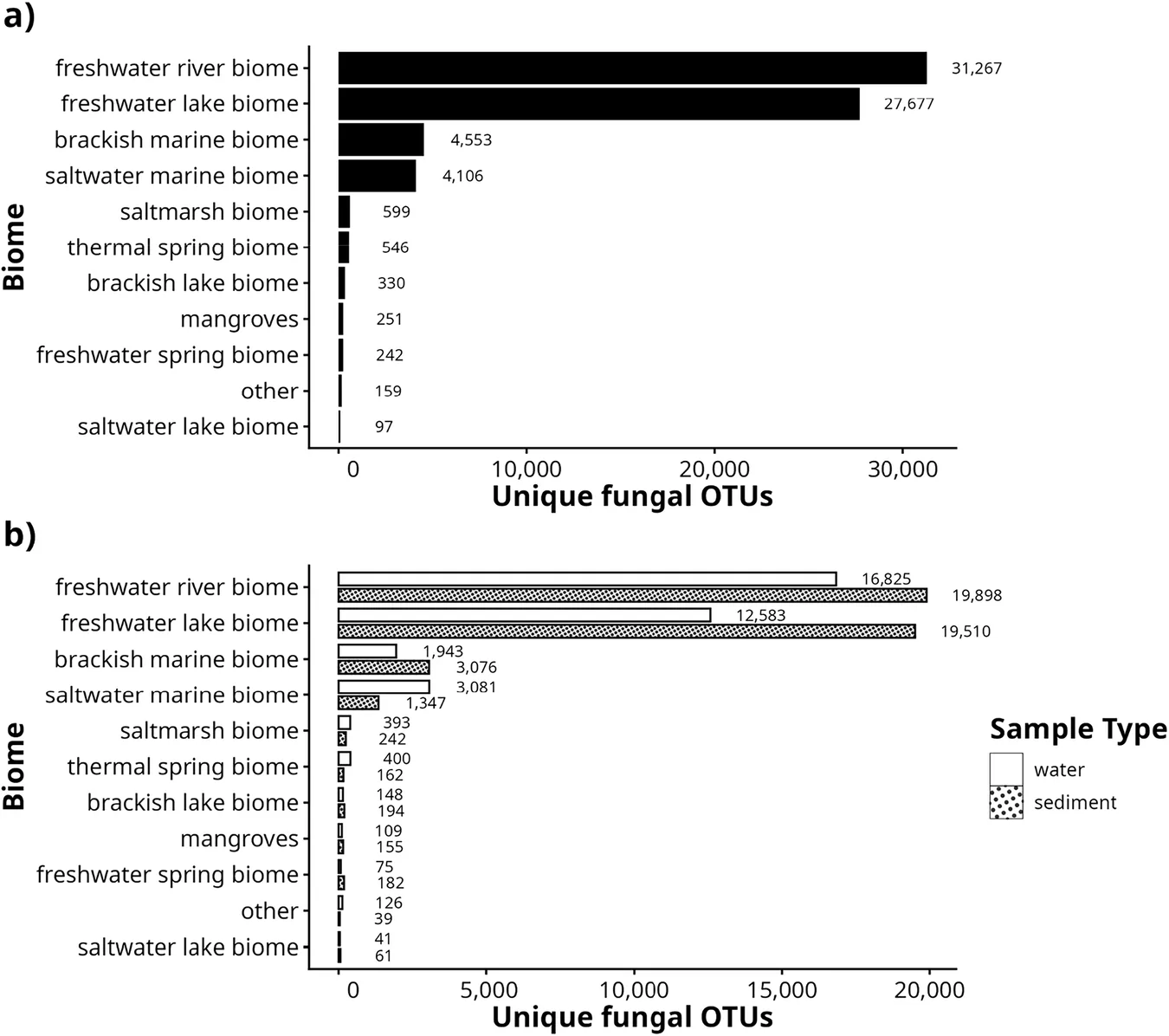
(**a**) number of unique fungal OTUs detected in all samples of each biome combined; (**b**) number of unique fungal OTUs detected in sediment and filtered water samples from each biome.

Among the sampled biomes, fungal sequences were detected in 1,026 freshwater lakes, 752 freshwater rivers, 276 saltwater marine, 153 brackish marine, 10 saltwater lakes, 9 brackish lakes, and 8 saltmarsh samples. All samples from mangroves, thermal springs, and freshwater springs contained fungal OTUs. Freshwater rivers contained the most fungal reads (983,828 reads; 31,267 unique OTUs), followed by freshwater lakes (879,797; 27,677), saltwater marine biome (108,936; 4,106), and brackish marine biome (70,039; 4,553) (Fig. 7a). From other biomes, a total of 22,807 fungal sequences (2,224 OTUs) were detected. In 7 biomes the number of fungal OTUs was larger in sediments than in water, while for the others, the OTUs detected from water outnumbered the sedimentary OTUs (Fig. 7b).

### Fungal taxonomic coverage

**Fig. 8:**
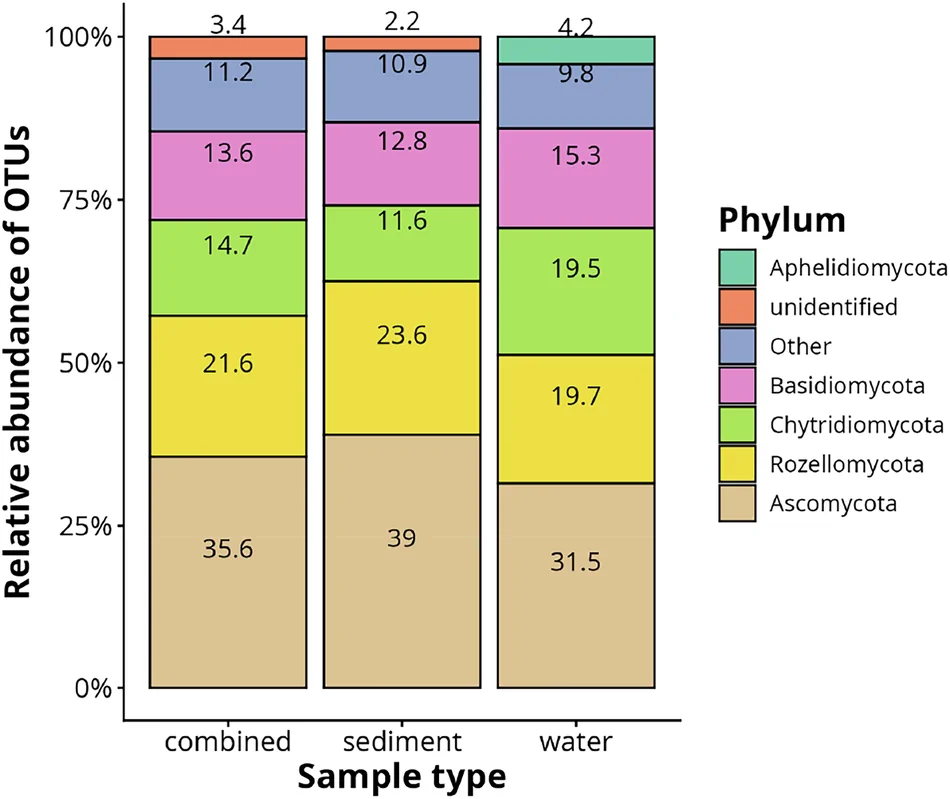
Stacked barchart presenting the relative abundance of the 5 most abundant phyla’s OTUs given in percentages.

Among the high-quality fungal OTUs detected, the phylum Ascomycota was the most abundant, comprising 35.6% (17,858 OTUs) of all fungal OTUs across combined samples (Fig. 8). This was followed by Rozellomycota (21.6%; 10,823 OTUs), Chytridiomycota (14.7%; 7,374 OTUs), and Basidiomycota (13.6%; 6,821 OTUs) (Fig. 8).

Clear differences in phylum-level composition were observed between sediment and water samples. In both environments, Ascomycota and Rozellomycota were dominant, however, their relative abundances differed. In sediments, Ascomycota accounted for 39.0% (13,300 OTUs) and Rozellomycota for 23.6% (8,046 OTUs), whereas in water samples these values were lower, at 31.5% (8,294 OTUs) and 19.7% (5,168 OTUs), respectively (Fig. 8). In sediment samples, Rozellomycota was followed by Basidiomycota (12.8%; 4,363 OTUs) and Chytridiomycota (11.6%; 3,962 OTUs) (Fig. 8). In contrast, in filtered water samples, Chytridiomycota (19.5%; 5,120 OTUs) closely followed Rozellomycota, with Basidiomycota ranking next (15.3%; 4,017 OTUs) (Fig. 8).

**Fig. 9:**
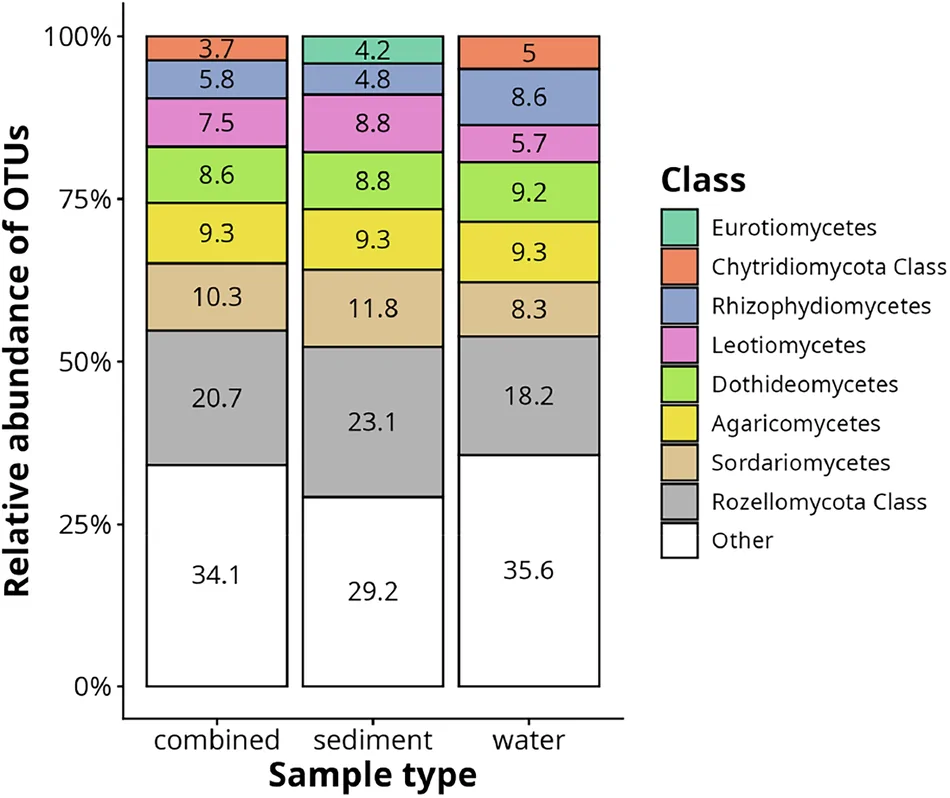
Stacked barchart presenting the relative abundance of the 7 most abundant classes’ OTUs given in percentages.

At both the class and order levels, unidentified Rozellomycota taxa represented the most abundant groups. When all samples were combined, these accounted for 20.7% (10,381 OTUs), with higher proportions in sediments (23.1%; 7,883 OTUs) than in water (18.2%; 4,796 OTUs). Among identified classes, Sordariomycetes was the most abundant overall (10.3%; 5,164 OTUs) and in sediment samples (11.8%; 4,043 OTUs), followed by Agaricomycetes (9.3%; 3,169) in sediments. Agaricomycetes (9.3%; 2,437) was the most abundant identified class in water, followed by Dothideomycetes (9.2%; 2,426) in water samples (Fig. 9).

**Fig. 10:**
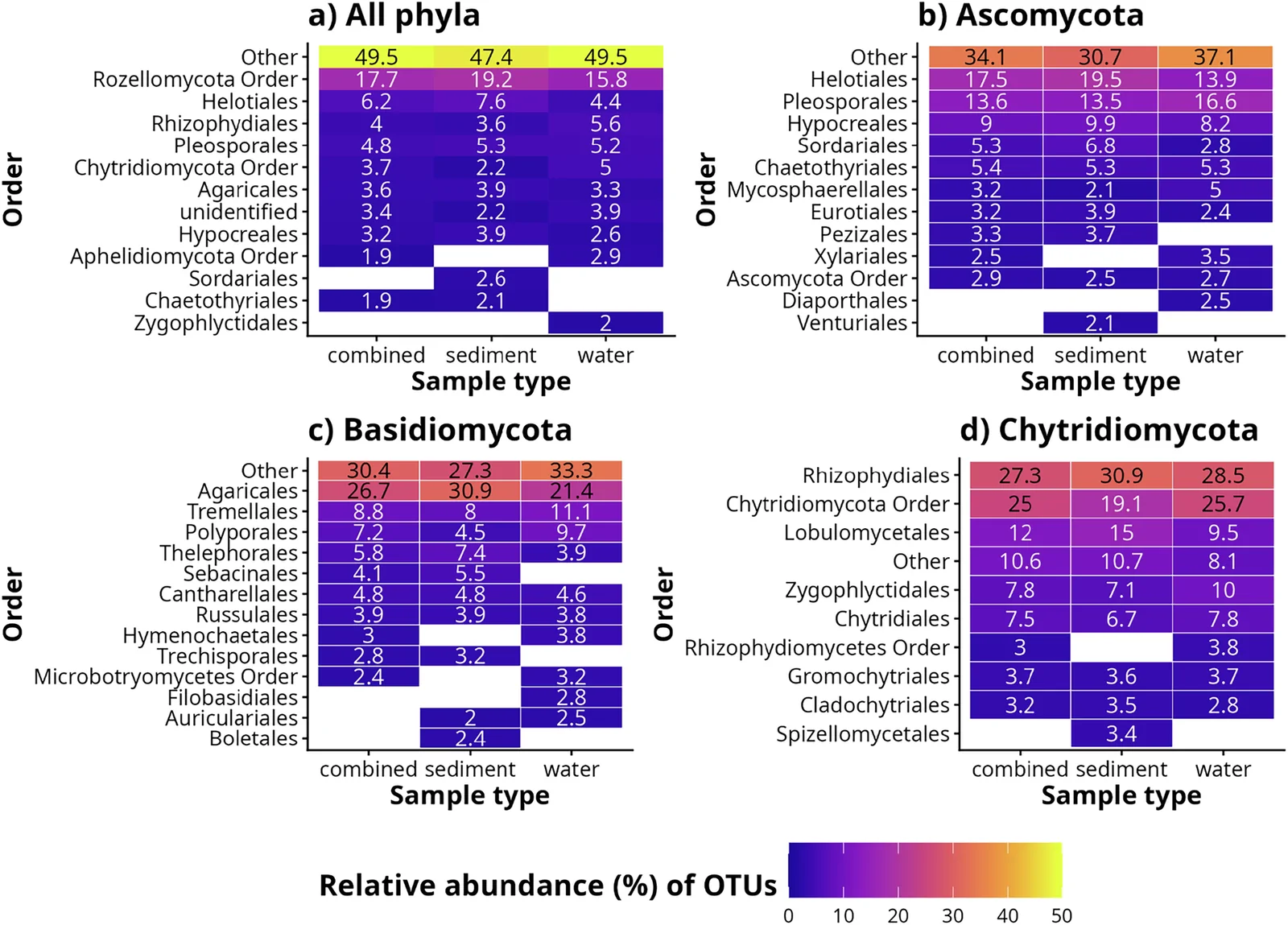
Heatmaps depicting the most OTU-abundant orders’ relative abundance in both sample types combined and separately. (**a**) Heatmap depicting the relative abundance of the most OTU-abundant orders overall given in percentages of all fungal OTUs; (**b**) heatmap depicting the relative abundance of Ascomycota orders given in percentages of Ascomycota OTUs; (**c**) heatmap depicting the relative abundance of Basidiomycota orders given in percentages of Basidiomycota OTUs; (**d**) heatmap depicting the relative abundance of Chytridiomycota orders given in percentages of Chytridiomycota OTUs. Names on the Y-axis ending with “Order” are unidentified orders of that specific taxon.

At the order level, unidentified Rozellomycota orders remained the most abundant across all samples (17.7%; 8,882 OTUs), dominating both sediments (19.2%; 6,571 OTUs) and filtered water samples (15.8%; 4,143 OTUs) (Fig. 10a). Helotiales was the most abundant order overall (6.2%; 3,132 OTUs) and in sediments (7.6%; 2,591 OTUs) (Fig. 10a). In contrast, in water samples, Rhizophydiales was the most abundant order (5.6%; 1,461 OTUs), exceeding Helotiales (4.4%; 1,151) in relative abundance (Fig. 10a). Helotiales comprised the largest subset of Ascomycota OTUs (17.5% of Ascomycota OTUs), and was subsequently the most abundant Ascomycota order in sediments (19.5%) (Fig. 10b). The most abundant Ascomycota order in water samples was Pleosporales (16.6%) (Fig. 10b). Agaricales accounted for 26.7% of Basidiomycota OTUs, making it the most abundant Basidiomycota order in both sediment (30.9%) and water (21.4%) samples (Fig. 10c). Among the chytrid OTUs, Rhizophydiales dominated, making it the most abundant in all samples combined (27.3%), sediments (30.9%) and water (28.5%) (Fig. 10d).

## Usage Notes

The FunAqua dataset is designed for broad reuse in fungal ecology, biogeography, and comparative biodiversity studies. Users should be aware of the following considerations when working with the data:

1. While most samples achieved adequate coverage for community-level analyses, samples with low sequencing depth may underestimate fungal OTU richness. We recommend filtering or rarefying samples or accounting for the sequencing depth in a way appropriate to the research question.
2. OTUs were clustered at 98% similarity, which approximates species-level resolution for ITS sequences across various fungal groups. However, it may merge closely related taxa, e.g., in some groups of filamentous Ascomycota, or split variable species, for example, in chytrids.
3. Our dataset does not distinguish between actively growing aquatic fungi, organisms inhabiting particles fallen into water or deposited spores. Therefore, caution is needed when drawing conclusions regarding the activity, lifestyle, traits or ecological functions of these fungi in water and sediments.
4. While the vast majority of the fungal kingdom is surveyed mainly based on the ITS marker, chytrids, aphelids, rozellids, and microsporidians mainly rely on SSU and/or LSU markers. Consequently, the diversity in these groups may be underestimated in ITS-based surveys. Although much of the taxonomic information from SSU and LSU markers has been transferred to long-read datasets through phylogenetic analyses in EUKARYOME, taxa within these early-diverging fungal lineages remain associated with greater taxonomic uncertainty.
5. The sampling locations are biased towards Europe. This is largely due to the project’s voluntary nature. At the time of publishing, additional sampling efforts are in process to cover underrepresented regions, and the dataset will be updated after the new samples are analysed.

We encourage researchers to contact the corresponding authors prior to large-scale reuse to facilitate collaboration and ensure the most up-to-date metadata are utilized. To promote transparent and fair usage, this dataset is tagged with Data Reuse Information (DRI)^45^ as described in the metadata files.

## Supplementary information

Supplementary Information

## Data Availability

The eight main dataset files are accessible on Zenodo at https://doi.org/10.5281/zenodo.20509884^36^, and the sequencing data can be obtained at the European Nucleotide Archive (ENA)^37^ under BioProject PRJEB104083 (Run Accessions ERR15968726 – ERR15971436). All data are currently under embargo and will be publicly released upon publication of this manuscript.

## Code availability

The R pipeline used to produce the majority of the technical validation steps covered under sections “Sampling coverage”, “Sequencing depth” and “Fungal Taxonomic coverage” is available on Zenodo together with the dataset. The code is accompanied by a RMarkdown document which produces a semi-interactive HTML output for data exploration.
